# A phenomenological study on psychological resilience among medical vocational college freshmen

**DOI:** 10.3389/fpsyt.2026.1816774

**Published:** 2026-04-30

**Authors:** Zhefu Zhang, Chunxiao Xu, Lipin Ye, Yue Yang, Ying Mao, Wan Cheng, Qiyuan Huang

**Affiliations:** 1 School of Public Health and Health Management, Fujian Health College, Fuzhou, China; 2 School of Nursing, Fujian Health College, Fuzhou, China; 3 Fujian Maternity and Children’s Hospital, Fuzhou, China; 4 School of Nursing, Wenzhou Medical University, Wenzhou, China; 5 School of Nursing, Xuzhou Medical University, Xuzhou, China

**Keywords:** medical vocational college freshman, mental health, phenomenological study, psychological resilience, qualitative research

## Abstract

**Background:**

Medical vocational college freshmen face severe challenges to their psychological resilience from various stressful events upon their enrollment. This qualitative study aimed to explore the authentic experiences and intrinsic characteristics of psychological resilience among medical vocational college freshmen.

**Methods:**

The study employed a descriptive phenomenological design. A purposive sample of 24 medical vocational college freshmen was recruited as participants. Semi-structured interviews were conducted to collect data between January 2025 and February 2025. The interviews were transcribed verbatim and analyzed using the Colaizzi descriptive analysis method.

**Results:**

Data analysis identified nine subthemes falling into three macrothemes: (a) Challenges: The Erosion of Psychological Resilience, describing how freshmen’s psychological resilience is eroded when they face difficulties in adapting to college life; (b) Support: The Recovery of Psychological Resilience, focusing on how freshmen regain resilience through internal and external support; (c) Cognition: The Maintenance of Psychological Resilience, explaining the factors that promote the sustained development of freshmen’s psychological resilience.

**Conclusion:**

Freshmen face pressures in academics, interpersonal relationships, and self-management. Family and peer support, together with personal growth, contribute to resilience recovery. Educators should employ cognitive restructuring, experiential learning, and other strategies to help maintain their psychological resilience.

## 1 Introduction

College life is a transitional process for students to adapt to changes as they move from campus life to social life. For new students entering college, they are adapting to changes in terms of identity, academic life, and psychology. Therefore, the first year of college is typically a high-risk growth stage for students to experience mental health disorders (1). A previous study showed that 35.3% of first-year college students suffer from mental health disorders, among which the incidence of mental health disorders in the past year is as high as 31.4% (2). Mental health disorders during the enrollment period not only cause significant psychological distress to college students during this major life transition but also correlate with declining academic performance, poor health, and suicidal thoughts or behaviors (3, 4). These adverse outcomes are particularly prevalent among medical students, who encounter relatively greater stress and challenges (5). To this end, the World Health Organization (WHO) World Mental Health International College Student (WMH-ICS) project advocates for attention to college students’ mental health and the early identification of unmet mental health service needs among the college student population (6).

Psychological resilience has been defined as “a process of good adaptation in the face of adversity, trauma, tragedy, threats or other significant stressors such as family and relationship problems, serious health problems or financial problems” (7). It is an important indicator for assessing medical college students’ adaptability (8).

Evidence indicates that psychological resilience is crucial for medical students. It helps them withstand the impact of stress, overcome challenges in both academic coursework and clinical practice, and prepare for their future professional roles (9). Therefore, exploring the cultivation and development of psychological resilience among new medical college students in a prolonged high-stress environment helps them better cope with the risks and challenges in the process of their growth.

Existing studies mainly explored the psychological resilience of medical students from individual intrinsic characteristics and the external environment. The individual’s internal self-cognition and coping ability are the core supporting forces that determine psychological resilience. Zhang et al. (10) found that psychological resilience mediates the relationship between professional identity and learning burnout. Specifically, the degree of medical students’ identification with their professional roles affects their psychological resilience, which in turn exerts a positive impact on their learning coping ability. Kumar et al. (11) argued that a high level of psychological resilience enables students to gain greater autonomy, which in turn allows them to adopt more positive coping strategies to alleviate daily stressors. The external environmental factors in the growth of medical students influence the cultivation of psychological resilience by providing support or exerting pressure. Arshad et al. (12) noted that perceived social support has a positive impact on the psychological resilience of medical students. Some potential family environment factors, including parental marital status and birth order, significantly influence students’ psychological resilience. Yang et al. (13) reported that psychological resilience plays a mediating role between academic stress and employment anxiety. They emphasized that appropriately alleviating academic stress during students’ transition to the workplace is of great significance for maintaining their mental health. However, current research on psychological resilience mostly focuses on the analysis of its mediating effects, while paying insufficient attention to the complexity and multidimensional characteristics of psychological resilience itself (14, 15). Furthermore, existing research on the psychological resilience of medical students has predominantly focused on undergraduate (16, 17). To the best of our knowledge, few studies have examined the psychological status of medical vocational college freshmen. This represents a significant gap, as vocational medical education in China serves a distinct purpose: its curricula are shorter and more intensely focused on the practical skills required in primary healthcare settings (18). These structural and contextual differences are likely to engender unique challenges, adaptive processes, and manifestations of psychological resilience, which cannot be reliably extrapolated from studies on undergraduate students (19). Given that the lived experience of psychological resilience among medical vocational college freshmen is still unclear, investigating this population will better inform strategies for the sustainable development of the human resources essential to the primary public health service sector.

Herrman (20) defined resilience as the capacity for positive adaptation, or the ability to maintain or regain mental health, despite experiencing adversity. It delineates the dynamic process of psychological resilience in individuals following adversity, including subsequent recovery, and maintenance. This framework provides guidance for the in-depth exploration of resilience experiences in this study. To our knowledge, there appears to be insufficient evidence to clarify the psychological resilience experiences of medical vocational college freshmen when coping with difficulties in adapting to university life. Only one study has examined the relationship between negative emotions and social functioning among Chinese medical vocational college students. However, their psychological resilience has not yet been explored in detail (21). Additionally, there is still a lack of evidence for medical educators regarding the psychological adaptation process of this group when they first enter college. This severely limits researchers’ ability to tailor reliable psychological resilience training programs for them. Therefore, we posed the research question: What are the lived experiences of psychological resilience among medical vocational college freshmen during their transition to university? To address these questions, this study aimed to explore the authentic experiences and intrinsic characteristics of psychological resilience among medical vocational college freshmen during their transition to university, thereby providing guidance for the development of effective intervention strategies.

## 2 Methods

### 2.1 Design

This study followed the descriptive phenomenological method of Colaizzi (22). It provides a phenomenological approach to understanding the psychological resilience of medical vocational college freshmen by exploring their feelings, experiences, and perceptions. During this study, we followed the standards for consolidated criteria for reporting qualitative studies (COREQ): a 32-item checklist (Additional file 1) (23).

### 2.2 Participants

Purposive sampling was used to recruit participants from one demonstrative public medical higher vocational college located in Fujian, China, between January 2025 and February 2025. To reduce confounding bias, maximum differential sampling was employed, with each major as the stratification factor. Allocation quotas for interviewees within each stratum were determined proportionally to ensure balanced representation of students across medical majors. With the assistance of major counselors, we provided students with research information, including an invitation letter, study purpose, eligibility criteria, potential risks, participation requirements, confidentiality and consent rights, withdrawal rights, and researchers’ contact information. Inclusion criteria were as follows: (1) full-time freshmen enrolled in medical vocational programs; (2) voluntary participation after explicit explanation of the study purpose and procedures. Exclusion criteria comprised: (1) students majoring in non-medical fields; (2) students repeating the first year due to study suspension or leave of absence (including military service), and international students.

### 2.3 Data collection

Data were collected through face-to-face semi-structured interviews, which were conducted in an independent, quiet, and private campus psychological counseling room at the participants’ convenience. To explore the core objective of how psychological resilience manifests among medical vocational college freshmen as they cope with challenges in adapting to college life, we designed a semi-structured interview guide. It was initially developed by the researchers (ZZ, QH) based on the research objectives, resilience theory (24), and field observation, then validated by three senior experts in qualitative research. The interview guide was pretested with two participants to ensure its appropriateness and feasibility. The pretest data were not included in the final analysis (25). The final version of the interview guide is shown in **Table 1**.

**Table 1:** The interview guide.

1. How do you feel after entering college?
2. What challenges did you encounter in adapting to college life? How did you overcome these challenges?
3. What factors can help you remain resilient in adversity?
4. Is there any way to help yourself recover resilience from adversity? If so, how do you do it?
5. Do you have any suggestions regarding the development and maintenance of psychological resilience during college life?

To ensure consistency in the interview process, each interview was conducted by the same researcher (ZZ). He is a master of psychological nursing with a strong background in qualitative research, extensive experience in college students’ psychological work, and excellent communication skills. We confirmed in advance that none of the participants had a prior relationship with the researcher, and all participants signed informed consent forms before the formal interview. During the interview, we adopted strategies to facilitate the process, including breaking deadlocks, building trust in relationships through both verbal and nonverbal words, and encouraging the participants to share their personal experiences and provide additional comments on the topic. The researcher tried his best to listen to the participants and observe at all times, adjust the interview rhythm, complete records, and maintain objectivity and neutrality. Field notes recording the participants’ facial expressions and body language as well as the researcher’s reflections were collected. Audio recordings were maintained throughout the interviews. The interviews were conducted in the Chinese, with a duration ranging from 30 to 50 minutes.

Data collection was pursued until the thematic content reached data saturation (26). After analyzing the transcripts of 22 interviewees, the research group identified that no further new codes or themes had emerged from the data. To confirm that data saturation had been achieved, two additional participants were interviewed. There were still no new categories emerging from the transcripts, indicating that data collection could be terminated.

### 2.4 Data analysis

The recording data were transcribed verbatim within 24 hours after the data collection. Data analysis was performed using the Colaizzi descriptive phenomenological analysis framework (22), which comprised seven analytical steps: (a) Audio recordings and written materials were transcribed and checked one by one by the first author (ZZ) to ensure completeness and accuracy. (b) Two researchers (ZZ, QH) listened to the recordings to gain an overall understanding of the phenomena. They independently read the transcripts multiple times to identify and extract significant statements. (c) The same two researchers marked, labeled, and coded recurring themes in the data. Before formal coding, the research team developed a clear coding manual that included operational definitions of psychological resilience and illustrative examples. Any significant statements regarding the psychological resilience among medical vocational college freshmen when coping with challenges in adapting to college life were extracted and identified. (d) Preliminary coded themes were compiled and organized by the research team for in-depth analysis and reflection. (e) Organization of each significant statement into meaningful units and sub-themes. (f) Similar viewpoints were further abstracted and refined into core themes by the research team. These themes were closely linked to the challenges encountered or the psychological resilience demonstrated during the freshman transition period. Each theme was thoroughly elaborated upon and explained. (g) Feedback on the results was provided to participants to ensure the accuracy and reliability of the findings. An example of the Colaizzi’s analysis is presented in **Table 2**.

**Table 2:** Example of the Colaizzi’s analysis.

Quotation	Interview number	Code	Sub-theme	Theme
*My older brother often advised me, helping me distinguish between what was important (setting goals and working diligently) and what was optional (letting things develop naturally).*	16	Support: Family support and guidance (from older brother)	Primary Support: Family as a Source of Emotional Solace and Encouragement	Support: The Recovery of Psychological Resilience
*I didn’t adapt well at the beginning. Whenever I felt sad, I would call my mom. After venting to her, I felt much better.*	11	Support: Family support of emotional solace (from parents)	Primary Support: Family as a Source of Emotional Solace and Encouragement	
*When I first started school, I felt very lonely. But later, I became close friends with a classmate who helped me get through the hardest times.*	7	Support:friendship that helps overcome loneliness and difficult times	Secondary Support: Peers as a Network for Emotional and Cognitive Assistance	
*After experiencing helplessness, I realized I had to become independent and stop relying on others for help. I started learning how to solve problems on my own*.	12	Self-support: Recognizing the need for independence and initiating self-directed problem solving.	Deep Support: Independence and Growth as Internal Resilience Drivers	

To enhance the breadth and transparency of the analysis, the research team also documented their critical reflections throughout the process. All preconceived notions regarding college students’ psychological resilience were set aside. During the theme-generation phase, we ensured the validity and reliability of the overarching themes through constant comparison and critical reflection until consensus was reached on the coded data for all relevant themes.

### 2.5 Study rigor

To ensure the rigor of this study, credibility, dependability, confirmability, and transferability were controlled throughout the research process (27). Credibility was ensured by investigator triangulation and peer debriefing (28). Ambiguities or controversies related to themes and sub-themes were thoroughly reviewed by three educational psychology researchers. The researchers tried to avoid confirmation bias, i.e., not supporting their conclusions when interpreting the data and not excluding any results that contradict their opinions. Final themes and sub-themes were finalized at the end of the peer review meeting. To enhance reliability, all stages of the study were reported in detail in accordance with the COREQ checklist to allow accurate judgments of our research procedures during peer review. Confirmability was ensured by member checks and searching for opposing evidence. All members, including participants, researchers, and peer experts, had the chance to explore and share their perspectives on the process and results of this study. Additionally, detailed quotations allow readers to assess the applicability of the categories in their contexts, thereby ensuring transferability.

### 2.6 Ethical consideration

Ethical approval was obtained from the Biological and Medical Research Ethics Committee of the first author’s institution before the study commenced. Before signing the written informed consent form, participants were fully informed of the purpose, process, and potential benefits of the study, as well as their right to withdraw at any time, and consented to audio recording. All audio recordings were saved in a password-protected computer until all interviews had been transcribed and verified against the recordings.

## 3 Results

A total of 25 students were recruited, and 24 participants were interviewed face-to-face. One student withdrew from the study due to illness prior to the formal interviews. The participants’ basic sociodemographic data were described using frequencies, percentages, and means are presented in **Table 3** and further detailed in **Supplementary Table 1**.

**Table 3:** Participant characteristics (*N* = 24).

Variables	Total (*N* = 24)
Age (year)	
Mean (*SD*)	18.08 ± 0.65
Gender *N* (%)	
Male	10 (41.67%)
Female	14 (58.33%)
Major *N* (%)	
Health Management	7 (29.17%)
Medical Nutrition	5 (20.83%)
Medical Imaging	2 (8.33%)
Rehabilitation Medicine	3 (12.50%)
Health Information Management	3(12.50%)
Preventive Medicine	4 (16.67%)
Student leadership position *N* (%)	
Yes	7 (29.17%)
No	17(70.83%)
Family Residence Location *N* (%)	
Urban Area	10 (41.67%)
Rural Area	14 (58.33%)
Frequency of Contact with Family *N* (%)	
Once a Day or More	5 (20.83%)
2–3 Times a Week	7 (29.17%)
Once a Week	7 (29.17%)
1–2 Times a Month	4 (16.67%)
Barely Contact	1(4.17%)

**Figure 1:**
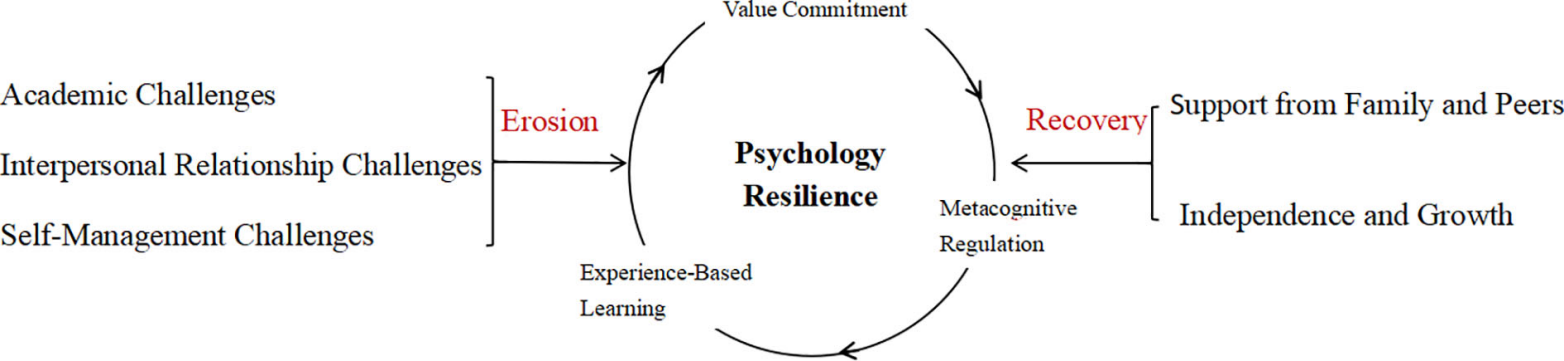
Thematic map of the psychology resilience.

Using Colaizzi’s phenomenological method, three themes were extracted from the data. These themes illuminate the psychological resilience experiences of medical college freshmen in coping with the difficulties of university adaptation. The first theme, “Challenges: the erosion of psychological resilience”, explored how adaptation difficulties undermine the psychological resilience of medical college freshmen. The second theme, “Support: recovery of psychological resilience”, focuses on describing how freshmen recover resilience through support from themselves and others. The third theme, “Cognition: maintenance of psychological resilience”, explains the factors that promote the maintenance of psychological resilience among freshmen. The frequency report table of research themes in this study is shown in **Supplementary Table 2**. We created a thematic map to reflect the manifestation of psychological resilience among medical vocational college freshmen during the admission adaptation process (**Figure 1**).

### 3.1 Challenges: the erosion of psychological resilience

Transitioning to university life involves a significant lifestyle transformation, and not all students can adapt swiftly to this novel environment. For freshmen enrolled in medical vocational education programs, in particular, college admission presents a notable challenge. The marked disparities in learning rhythms, interpersonal interactions, and living environments collectively amplify their difficulties in adaptation, gradually eroding their psychological resilience.

#### 3.1.1 Academic challenges: pressure-induced anxiety and cognitive overload

Compared to undergraduate medical education, medical vocational education in China is characterized by a shorter duration and a more intensive academic workload. The curriculum prioritizes the integration of theoretical knowledge and practical application, with academic performance evaluated based on students’ comprehensive competencies—posing substantial demands on the learning capabilities of medical vocational freshmen. As Participant 5 noted:

*“Upon enrollment, the medical curriculum was extremely extensive, accompanied by intense pressure. Additionally, the teaching methodologies and learning content diverged significantly from what I was accustomed to. We had to balance theory classes, practical sessions, clinical rotations, and online courses—this placed a substantial strain on our overall learning capacity.”*

This heavy academic burden often leads to cognitive overload, triggering adaptation-related stress and anxiety. Participant 1 elaborated on this experience:

*“University homework differed entirely from what we had encountered in high school. It was no longer limited to textbook exercises; we also had practical training and independent learning tasks, which I struggled to adapt to. With a dense course schedule and end-of-semester exams looming, I frequently felt anxious and unable to keep pace.”*

Students must rely on their psychological resilience to cope with these persistent pressures. During peak periods of stress (e.g., quizzes, assignment deadlines), students often reach the limits of their resilience, leading to a sense of emotional collapse. As Participant 3 described:

*“Ordinarily, I could persevere or distract myself from stress, but when faced with quizzes or approaching deadlines, I found it overwhelming and even considered giving up.”*

#### 3.1.2 Interpersonal relationship challenges: collective life conflict and individual isolation

University social networks expand considerably, extending beyond the classroom to encompass clubs, student unions, dormitories, and other collective settings. Active participation in these groups and engagement in collective activities are critical for students’ holistic development. However, for some students—especially those with introverted personalities—this social expansion becomes a barrier, impeding their initial adaptation. Participant 15 shared:

*“I saw others joining clubs and student unions, and I genuinely wanted to participate too. But I was quite introverted and had few hobbies, so I feared being excluded. In the end, I just decided to give up.”*

Notably, many interviewees were experiencing collective living (e.g., dormitory life) for the first time—a stark contrast to family life. Adapting to dormitory dynamics and resolving interpersonal conflicts have emerged as prominent challenges, generating feelings of loneliness and psychological stress that further erode resilience. Participant 11 reflected:

*“I used to live with my parents, so adapting to group living has been notably challenging. My roommates had different living habits, so conflicts were inevitable. Even though I lived in a group, I felt deeply lonely inside.”*

#### 3.1.3 Self-management challenges: the struggle with disordered rhythms

University grants students greater autonomy, which creates opportunities for growth but also tests their self-management capabilities. Without the strict supervision of teachers or parents, students must independently manage their time and tasks. Failure to do so often traps them in a vicious cycle of procrastination and anxiety—resulting in a paradox of “having freedom but feeling uneasy.” Participant 3 explained:

*“University has given me far more control over my time. I no longer felt a sense of urgency, and my study pace slowed dramatically. This was a real test of self-discipline. I knew this state was detrimental to my development, but I still could not help but relax.”*

Such deficits in self-discipline lead to a lack of initiative and planning when addressing academic and life tasks, hindering personal growth. Participant 9 contrasted this with high school experiences:

*“In high school, I had a fixed schedule—specific tasks for specific times. Once I entered university, with no one supervising me, my self-discipline deteriorated. I lowered my standards for myself and lacked motivation and structure in my work.”*

### 3.2 Support: the recovery of psychological resilience

Psychological resilience is not merely an individual trait but one nurtured by multilevel support systems. For medical vocational freshmen, these systems include primary family support, secondary peer support, and deep internal support derived from independence and growth.

#### 3.2.1 Primary support: family as a source of emotional solace and encouragement

Family serves as the most crucial external support for maintaining psychological resilience among medical vocational freshmen. Despite potential generational gaps that may prevent parents from fully comprehending the specific challenges their children face (and corresponding solutions), the emotional comfort, trust, and encouragement they provide remain indispensable. Participant 19 noted:

*“Both my parents worked in other cities. When I encountered difficulties, they might not have fully understood the pressures of university life, but they always said, ‘It is okay—take your time. We are here for you.’ Their reassurance put my mind at ease.”*

Family support is not limited to intergenerational bonds; family members with analogous experiences (e.g., university education) can offer deeper emotional resonance. By discussing adaptation challenges with students during difficult periods, these individuals help alleviate anxiety and strengthen resilience. For example, Participant 16,whose parents worked away and rarely visited—cited his older brother (a university graduate and working professional) as a key support:

*“My older brother was incredibly important to me. He had gone to university and worked in society for years, so he could empathize with my struggles. That feeling of being understood … He often advised me, helping me distinguish between what was important (setting goals and working diligently) and what was optional (letting things develop naturally). After talking to him, I regained my confidence.”*

#### 3.2.2 Secondary support: peers as a network for emotional and cognitive assistance

Peer support is the most accessible resource for freshmen, both spatially and temporally. Emotionally, freshmen newly arrived on campus often experience loneliness; peer companionship and active listening effectively mitigate negative emotions, foster a sense of belonging, and accelerate their integration into campus life. Participant 7 shared:

*“When I first started school, I felt very lonely. But later, I became close friends with a classmate who helped me get through the hardest times.”*

Participant 10 emphasized the stability of long-term peer bonds:

*“I have a childhood friend—we grew up together. No matter where we study, as long as she was with me, everything felt okay. I could share anything with her, and I was truly grateful to her.”*

Cognitively and behaviorally, peers—who have shared similar experiences—are more likely to empathize and offer diverse perspectives, broadening students’ cognitive horizons and enabling them to approach challenges with greater openness. When freshmen contemplated giving up, peer encouragement often motivated them to take proactive steps to solve problems. Participant 22 explained:

*“Only my friend could truly understand the problems I faced at university. She had been through similar things, so she could empathize and give valuable advice. I felt very lucky to have her.”*

Notably, some students exhibited a “support network compensation phenomenon”: the absence of family support led to increased reliance on peers, reflecting a unique social support compensation mechanism among this cohort. Participant 9 described this dynamic:

*“My parents were busy with work every day and did not understand my life. I had never had many friends, so I shared most of my thoughts with an online friend. We exchanged daily stories and messages—this was a form of spiritual companionship.”*

#### 3.2.3 Deep support: independence and growth as internal resilience drivers

Self-reflection following adversity is a pivotal opportunity for student growth and a key internal mechanism for sustaining psychological resilience. When facing challenges, students must not only rely on external support but also activate internal motivation: through reflection and action, they enhance their competencies, gradually develop the ability to solve problems independently, and thereby replenish their resilience while gaining a sense of empowerment. Participant 20 reflected on this transition:

*“At first, I was heavily dependent on my parents and friends—handing over all my problems to them to solve. But after entering university, I gradually realized that problems have to be addressed by myself. Life is something you live for yourself; others cannot truly help you, and you cannot rely on them forever. As I began to grow and take responsibility, I discovered that I had more strength than I thought—the strength to solve these problems on my own.”*

This shift from dependence to independence equips students to face future challenges with greater confidence and composure, leveraging their own abilities and wisdom to overcome obstacles. Importantly, medical vocational education distinguishes these students from other university cohorts by emphasizing resilience cultivation through practical experience. Participant 12 noted:

*“After experiencing helplessness, I realized I had to become independent and stop relying on others for help. I started learning how to solve problems on my own.”*

### 3.3 Cognition: the maintenance of psychological resilience

Individual differences in cognitive processes determine how effectively students maintain psychological resilience even after receiving support. Some students actively cope with difficulties and sustain high resilience following family communication, while others remain in poor mental states—even a single criticism can push their psychological defenses to the brink of collapse. Key cognitive factors promoting resilience include metacognitive regulation, experience-based learning, and value commitment.

#### 3.3.1 Metacognitive regulation: reconstructing negative cognitions into positive ones

Mindset transitions play a pivotal role in sustaining psychological resilience during psychological development and adaptation to complex environments. When facing adversity, shifting from a fixed mindset (e.g., “failure reflects my abilities”) to a growth mindset (e.g., “failure is an opportunity to improve”)—and reconstructing negative cognitions into positive ones—enables students to view setbacks as catalysts for growth rather than rejections of their competence. Participant 13 illustrated this shift:

*“Take my recent exam failure, for instance. In the past, I would have felt upset and thought, ‘I am stupid—I cannot learn this.’ But this time, I realized that dwelling on the past was useless. I started changing my mindset: it was okay if I did not perform well in one exam … Overall, I felt my resilience was growing.”*

This cognitive reconstruction fosters perseverance and a positive outlook, enabling students to remain resilient amid challenges. Participant 15 added:

*“At first, I was anxious and thought, ‘I cannot do this.’ But later, I told myself, ‘This is a challenge, and I can handle it. As long as I do not give up, there is always a way.’ I became more positive. “*

#### 3.3.2 Experience-based learning: leveraging personal and vicarious experiences

An experience-based learning system is a critical pillar for sustaining psychological resilience among medical vocational freshmen. When their resilience is tested by adversity, students draw on analogous past experiences and sustain their resolve through the conviction that “*If I succeeded before, I can succeed now.*” Participant 3 — a holder of a Level 10 dance certification—explained:

*“I had been dancing since childhood and persisted for years—that was a core belief of mine. When I faced problems I could not solve, I thought back to my dance training and how I had overcome similar difficulties. For example, when I first became a student representative, the workload overwhelmed me and made me want to quit. But I reminded myself: if I could persist through dance, I could persist then.”*

Vicarious experiences (e.g., those of seniors or peers) also play a vital role. For instance, senior students’ experiences help freshmen recognize that their challenges are not unique, reducing self-doubt and anxiety. Participant 10 noted:

*“Seniors’ experiences were incredibly helpful. Learning that they also faced dormitory conflicts like mine made me realize I was not a bad person—and I was not the only one going through this.”*

Peer success stories further serve as role models, inspiring confidence and motivation. Participant 23 shared:

*“When I struggled with difficult coursework, I sometimes talked to my roommates. Seeing them persevere gave me a lot of confidence—I did not want to give up either. I believed I could do it too.”*

#### 3.3.3 Value commitment: rational cost-benefit reflection and familial responsibility

Participants mentioned that they engaged in deliberate reflection on the costs and benefits of their efforts and exhibited a mechanism of value preservation and commitment toward their endeavors—an important factor in sustaining resilience. If students recognized that their years of academic investment could not be squandered, they chose to persevere to secure better future outcomes. Participant 1 stated:

*“I had studied for so many years, and this was the final stage of my education. While I was still adjusting to university life, this diploma would help me find a job. If I gave up and dropped out then, all those years of study would be wasted—I could not let that happen.”*

This value commitment also extended to others: gratitude and a desire to repay family members’ investments and sacrifices motivated students to persist. For example, Participant 2 — raised by his grandmother after his parents’ divorce, with his father working away — explained:

*“My grandmother was the reason I kept going. She had sacrificed everything for me; without her, I would not have been here studying. Sometimes I wanted to give up, but then I thought about all she had done over the years. I told myself I must finish university and find a good job to repay her.”*

This rational cost-benefit analysis, combined with a sense of familial responsibility, enables students to remain steadfast in their goals, sustain psychological resilience, and strive to overcome challenges to complete their education.

## 4 Discussion

This qualitative study explored the authentic experiences and intrinsic characteristics of psychological resilience among medical vocational college freshmen during their transition to college. The participants described the challenges they faced in terms of psychological resilience in academics, relationships, and self-management during their early college enrollment. They believed resilience could be restored through diverse support systems and personal growth. By utilizing cognitive regulation, experiential learning, and value commitment, they were able to maintain resilience. This study enhances understanding of freshmen’s psychological resilience and provides a reference for educators to develop targeted psychological support interventions.

This study found that the difficulties faced by medical vocational college freshmen during the enrollment stage are complex and diverse, which can lead to a certain degree of depletion and pose challenges to their psychological resilience. The findings are consistent with the risk factors associated with psychological resilience as conceptualized by Herrman (20). It emphasized that psychological challenges typically arise from poor interpersonal relationships and negative life events. Building on this theoretical foundation, the present study further clarified the specific challenges from academic pressure and the lack of self-management skills among vocational medical college freshmen may undermine resilience. These research findings not only validate this theoretical framework but also expand its application scope by clearly identifying contextually significant risk factors in the educational context. Participants attributed the difficulties they faced to academic, interpersonal, and self-management factors. First, the vast and highly specialized nature of the medical knowledge system results in an excessive academic workload, which brings pressure for adaptation and negative emotions to freshmen. This finding is similar to those of Gong et al. (29). These authors confirmed the mediating role of psychological resilience between academic stress and burnout, and suggested alleviating burnout by reducing academic stress and enhancing psychological resilience. Participants indicated that academic stress stems not only from knowledge overload but also from students’ poor adaptation to changes in learning modes and their own weak psychological resilience. Students with low psychological resilience tend to adopt a pessimistic attitude when facing setbacks and difficulties, which often prevents them from persisting in their goals and finding suitable coping strategies (29). On the other hand, moderate academic stress can also positively impact psychological resilience. Liu et al. (30) demonstrated a significant positive correlation between psychological resilience and academic stress. Therefore, during the freshman enrollment stage, medical educators should appropriately manage students’ academic stress. Some interviewees mentioned that during the mid-term and final exams, they also experienced peak academic pressure. This indicated that during these periods, implementing appropriate support measures can help enhance students’ psychological resilience.

Secondly, college freshmen face a relatively complex interpersonal environment, primarily reflected in their experience of living in a dormitory and participating in various forms of group social activities, which leads to adaptation difficulties and interpersonal conflicts. Zhang et al. (31) noted a positive correlation between psychological resilience and interpersonal relationships. Students with lower levels of psychological resilience lack initiative in social interactions (such as positive parent-child, teacher-student, and peer interactions) and are more likely to be troubled by interpersonal relationships. In this study, some participants reported fearing social exclusion and being unwilling to engage in excessive group interactions, which resulted in a certain degree of depletion of their psychological resilience. Erikson’s theory of psychosocial development explains this phenomenon by suggesting that if college students are unable to share their emotions and communicate effectively with others, they will experience emotional exhaustion (32). Participants described the critical role of a sense of belonging in the recovery of psychological resilience. Therefore, medical educators should, in the early stage of enrollment, serve more as “matchmakers” for freshmen’s interpersonal relationships, helping them establish a sense of belonging within the collective. Such freshman adaptation intervention deserves attention and validation in future research.

Finally, participants reported that after entering university, their self-management approach tended to undergo a passive transformation, resulting in a state of freedom yet confusion among students with poor self-management abilities. This finding is similar to that of Jing et al. (33), who pointed out that students with stronger self-management abilities are more confident, more optimistic, and have higher levels of resilience. In contrast, students with poor self-management abilities often lack initiative and planning in their studies and daily lives, making them more prone to reduced resilience. Therefore, we infer from the interview findings that medical educators can provide relevant guidance through self−management courses during the enrollment stage to help freshmen develop a positive self−management plan.

This study discovered that family, friends, and internal support influence the recovery of freshmen’s psychological resilience. Due to the generational gap, parents primarily provide emotional comfort within family support. Family members, especially cousins who share similar learning experiences, are better able to empathize and help students develop greater resilience. Zhang et al. (34) demonstrated that family support plays an important role in students’ psychological resilience, and students with insufficient family support found it more difficult to maintain positive emotions when facing adversity or challenges. In this study, some participants indicated that compared with family support, they were more willing to seek support from friends, and friend support could have a greater impact on their subjective initiative. This result is an intriguing phenomenon that stands in sharp contrast to the core idea of Chinese family education, which centers on the concept of “the family as the foundation of the state.” A plausible explanation is that, due to temporal and spatial convenience, peer relationships are more likely to foster a sense of belonging and connection, making freshmen feel closer to their peers (35). Particularly among medical students, who face significant academic pressure, the long-term mutual support and companionship among peers enable them to more readily seek a sense of belonging (36). This suggests that medical educators should attach importance to home-school communication and fully utilize the role of families in providing emotional support. They should also pay attention to the social connections between medical freshmen and their peers. In particular, during the school period, when students have insufficient contact with their families, a peer mutual support and growth system of “identification–support–growth” should be actively established. Jin et al.’s (37) study also advocated that helping college students establish positive peer relationships was beneficial for them to adopt effective coping mechanisms against unknown challenges. Some participants also indicated that as the adaptation period progresses, they gradually develop the ability to solve problems independently, thereby continuously restoring their level of psychological resilience. Resilience theory also highlights that individuals who can successfully recover from adversity are likely to experience a period of rapid post-adversity growth (20).

Psychological resilience is a dynamic adjustment process that enables individuals to cope with adversity, requiring them to maintain their balance independently. Herrman proposed that cognitive appraisal, positive interpretation of events and cohesive integration of adversity into self-narrative, play a significant role for individuals (20), and similar findings were observed in our study. Our participants shared, from multiple perspectives, strategies for maintaining psychological resilience, with cognitive restructuring emerging as a key strategy. Some participants reported that as they gradually recovered from adversity, a shift from negative to positive cognition occurred. This enables individuals to view setbacks as opportunities for growth, allowing them to face new challenges without giving up and to maintain a higher level of psychological resilience. Cognitive restructuring theory explains this phenomenon by suggesting that it changes students’ evaluation of stress perceptions, enabling them to cope with new stressors in a more positive state (38). Hastuti et al. (39) also confirmed that cognitive restructuring interventions mediated by group members could effectively enhance students’ psychological resilience. In addition, students also engage in self-reflection based on others’ or their own past experiences. When their resilience is tested by adversity, they often recall similar experiences to identify coping strategies, thereby reducing self-doubt and anxiety and maintaining a favorable state of resilience. Drawing on experiences as a form of learning allows students to acquire knowledge and skills for coping with adversity more quickly, leading them to view current challenges as “solvable problems” and thereby enhancing their psychological resilience (40). Milli’s (41) study also confirmed that group-based experiential learning interventions, which provide students with a specific framework for coping with adversity, can effectively enhance their psychological resilience. Therefore, medical educators can rely on senior students to organize experience-sharing sessions among freshmen, thereby expanding the channels through which new students can access adaptation-related experiences for university life. We also found that when freshmen coped with adversity, they tended to evaluate their input and output rationally, and a value commitment mechanism existed. It was an intriguing phenomenon that could originating from both internal and external sources, namely, students’ gratitude for the efforts of others. Self-worth theory explains these findings by suggesting that when students believe their parents have invested a great deal of effort in their studies, they tend to view academic success as a way to repay their parents’ efforts, thereby motivating themselves to study harder (42). This regulatory mechanism plays a crucial role in safeguarding students’ self-worth and maintaining their psychological resilience. Davey et al. (43) also demonstrated the same conclusion. Therefore, strengthening freshmen’s awareness of gratitude for the efforts of others and guiding them to develop the ability to evaluate input and output rationally is expected to help them maintain a positive mindset in the face of adversity, thereby achieving the dual goals of academic adaptation and psychological growth.

## 5 Limitations

This study had some limitations. First, the study exclusively recruited medical vocational college freshmen, excluding the perspectives of college counselors and family members. To provide a more comprehensive explanation of the psychological adaptation difficulties faced by new medical students during the enrollment period and formulate targeted coping strategies, it is essential for future research to incorporate the perspectives of more stakeholders. Second, due to ethical considerations, this study recruited participants through open recruitment. This may have resulted in our sample consisting primarily of students who were willing to share, discuss, or focus on psychological resilience. Some students who were potentially experiencing psychological distress may have been reluctant to participate. This lead to sampling bias. Finally, although we reached data saturation, our sample size was relatively small and the data collection sites were relatively limited. Given the limitations of the study setting and the variances in Chinese medical education systems, the transferability of the research conclusions still requires further verification across multiple regions. In addition, future longitudinal qualitative studies to collect participants’ psychological adaptation experiences across different college stages is expected to facilitate a more in-depth understanding of the long-term development of psychological resilience among medical vocational college students.

## 6 Conclusion

This study provides valuable information regarding the characteristics of psychological resilience among medical vocational college freshmen. The psychological resilience of these new students is confronted with challenges from stressors related to academics, interpersonal relationships, and self-management. Nevertheless, the insights from students emphasize the importance of external support and personal growth for the recovery and maintenance of psychological resilience. Medical colleges should be encouraged to establish a mental health support system that integrates family-school collaboration and peer mutual assistance, and to conduct research on psychological resilience intervention strategies with a focus on self-efficacy enhancement, cognitive restructuring, and experiential reflection.

## Data Availability

Due to the privacy of the participants involved in the study data, the datasets generated and/or analyzed in the study are not currently publicly available but are available from the corresponding authors of this study upon reasonable request.

## Supplementary material

Supplementary Table 1 Characteristics of participants.
