# Supplementary material for: A phenomenological study on psychological resilience among medical vocational college freshmen
Source: Front Psychiatry. 2026 Apr 30;17:1816774. doi: 10.3389/fpsyt.2026.1816774 (PMC13171832; doi:10.3389/fpsyt.2026.1816774)
Supplement: Supplementary Table 1 — Characteristics of participants. [file Table1.docx]

| **Supplementary Table 1. Characteristics of participants** | | | | | | |
| --- | --- | --- | --- | --- | --- | --- |
| **Participants** | **Age** | **Gender** | **Major** | **Student leadership position** | **Family Residence Location** | **Frequency of Contact with Family** |
| 1 | 17 | Male | Medical Imaging | No | Rural Area | 2-3 Times a Week |
| 2 | 18 | Male | Medical Imaging | No | Rural Area | 1-2 Times a Month |
| 3 | 18 | Female | Health Management | No | Urban Area | Once a Week |
| 4 | 18 | Female | Health Management | Yes | Rural Area | Once a Week |
| 5 | 19 | Male | Health Management | No | Rural Area | Once a Day or More |
| 6 | 19 | Male | Health Management | No | Rural Area | 2-3 Times a Week |
| 7 | 17 | Female | Health Management | Yes | Urban Area | 2-3 Times a Week |
| 8 | 18 | Female | Health Management | Yes | Rural Area | Once a Week |
| 9 | 19 | Female | Health Management | Yes | Rural Area | 2-3 Times a Week |
| 10 | 18 | Male | Preventive Medicine | No | Rural Area | 1-2 Times a Month |
| 11 | 18 | Female | Preventive Medicine | No | Urban Area | Once a Week |
| 12 | 18 | Male | Preventive Medicine | No | Urban Area | Once a Week |
| 13 | 19 | Male | Preventive Medicine | Yes | Urban Area | Once a Day or More |
| 14 | 17 | Female | Medical Nutrition | No | Rural Area | Once a Day or More |
| 15 | 18 | Female | Medical Nutrition | Yes | Rural Area | 2-3 Times a Week |
| 16 | 18 | Female | Medical Nutrition | Yes | Urban Area | Once a Day or More |
| 17 | 18 | Female | Medical Nutrition | No | Urban Area | 1-2 Times a Month |
| 18 | 18 | Male | Medical Nutrition | No | Rural Area | Once a Week |
| 19 | 18 | Female | Rehabilitation Medicine | Yes | Urban Area | 2-3 Times a Week |
| 20 | 19 | Male | Rehabilitation Medicine | No | Rural Area | 2-3 Times a Week |
| 21 | 19 | Female | Rehabilitation Medicine | No | Urban Area | Once a Week |
| 22 | 17 | Female | Health Information Management | Yes | Urban Area | Once a Day or More |
| 23 | 18 | Male | Health Information Management | No | Rural Area | 1-2 Times a Month |
| 24 | 18 | Female | Health Information Management | No | Rural Area | Barely Contact |
