# Supplementary material for: A phenomenological study on psychological resilience among medical vocational college freshmen
Source: Front Psychiatry. 2026 Apr 30;17:1816774. doi: 10.3389/fpsyt.2026.1816774 (PMC13171832; doi:10.3389/fpsyt.2026.1816774)
Supplement: Supplementary file 2 [file Table2.docx]

**Supplementary Table 2. The frequency table of categories reports**

| **Main themes** | **Sub-themes** |  | **Participants** |
| --- | --- | --- | --- |
| Challenges: The Erosion of Psychological Resilience | Academic Challenges: Pressure-Induced Anxiety and Cognitive Overload |  | P1,P3,P5,P6,P11,P12,P13,P15,P16,P20,P21,P24 |
|  | Interpersonal Relationship Challenges: Collective Life Conflict and Individual Isolation |  | P2,P6,P11,P15,P21 |
|  | Self-Management Challenges: The Struggle with Disordered Rhythms |  | P3, P9, P17, P19 |
|  |  |  |  |
| Support: The Recovery of Psychological Resilience | Primary Support: Family as a Source of Emotional Solace and Encouragement |  | P5, P13,P16, P19, P20 |
|  | Secondary Support: Peers as a Network for Emotional and Cognitive Assistance |  | P3, P4, P7,P9,P10,P22 |
|  | Deep Support: Independence and Growth as Internal Resilience Drivers |  | P1,P12, P20,P24 |
|  |  |  |  |
| Cognition: Sustaining Psychological Resilience | Metacognitive Regulation: Reconstructing Negative Cognitions into Positive Ones |  | P13, P15,P18 |
|  | Experience-Based Learning: Leveraging Personal and Vicarious Experiences |  | P3, P10, P17, P22 |
|  | Value Commitment: Rational Cost-Benefit Reflection and Familial Responsibility |  | P1,P2 |
